# Increase in intracellular PGE_2 _induces apoptosis in Bax-expressing colon cancer cell

**DOI:** 10.1186/1471-2407-11-153

**Published:** 2011-04-27

**Authors:** Lisenn Lalier, François Pedelaborde, Christophe Braud, Jean Menanteau, François M Vallette, Christophe Olivier

**Affiliations:** 1 Département de Biologie Oncologique, Centre de Lutte Contre le Cancer René Gauducheau, Bd J. Monod, 44805 Nantes, Saint Herblain Cedex, France; 2 Département de Recherche en Cancérologie, Université de Nantes, INSERM U892, 8 quai Moncousu, 44035 Nantes Cedex, France; 3 Laboratoire de Toxicologie, Faculté de Pharmacie, Université de Nantes, 1 rue Gaston Veil, 44000 Nantes, France

## Abstract

**Background:**

NSAIDs exhibit protective properties towards some cancers, especially colon cancer. Yet, it is not clear how they play their protective role. PGE_2 _is generally shown as the only target of the NSAIDs anticancerous activity. However, PGE_2 _known targets become more and more manifold, considering both the molecular pathways involved and the target cells in the tumour. The role of PGE_2 _in tumour progression thus appears complex and multipurpose.

**Methods:**

To gain understanding into the role of PGE_2 _in colon cancer, we focused on the activity of PGE_2 _in apoptosis in colon cancer cell lines.

**Results:**

We observed that an increase in intracellular PGE_2 _induced an apoptotic cell death, which was dependent on the expression of the proapoptotic protein Bax. This increase was induced by increasing PGE_2 _intracellular concentration, either by PGE_2 _microinjection or by the pharmacological inhibition of PGE_2 _exportation and enzymatic degradation.

**Conclusions:**

We present here a new sight onto PGE_2 _in colon cancer cells opening the way to a new prospective therapeutic strategy in cancer, alternative to NSAIDs.

## Background

Prostaglandins are implicated in a wide range of physiological and pathological pathways. Among these pathways, cancer occurrence and development is one of the most debated. It is undoubtable that NSAIDs use was shown to reduce the incidence of some cancers [1], among which colon cancer took the highest therapeutic advantage [1]. It is unclear, however, how NSAIDs play their protective role. At the tissue level, chronic inflammation is implicated in the development of cancers [2]. Proinflammatory prostaglandins play a role in tumour progression in many ways, namely cell proliferation, survival and migration, immunosuppression and angiogenesis [2]. The anti-inflammatory activity of NSAIDs is thus probably involved in their anti-cancer potency. Yet, at the cellular level, the mechanism by which NSAIDs exert their proapoptotic activity is not clear. PGE_2 _itself has been shown to play various roles in cell survival and proliferation (reviewed in [3]). PGE_2 _induces the activation of several pathways in cancer cells through its interaction with membrane receptors EP(1-4) [3], and nuclear receptors (PPARδ) [4], thereby promoting proliferation and survival. Besides, 15-PGDH, the enzyme responsible for its degradation, has been identified as a negative regulator of colon cancer progression [5]. Nevertheless, some models demonstrate a more complex role played by PGE_2_, since it induces cell death under some circumstances. Thus, it was shown that PGE_2 _could mediate both neuroprotection and neurotoxicity through the same EP2 receptor, depending on the conditions [6]. Huang and colleagues also demonstrated an EP2/EP4-mediated apoptotic role of PGE_2 _in fibroblasts [7]. Moreover, PGE_2 _was also shown to exert opposite effects on colon cancer cells proliferation through different signalling pathways depending on the range of its concentration in the cell culture [8].

Strikingly, although NSAIDs modulate the production of several prostaglandins, their inhibiting efficiency is classically monitored by the sole measurement of PGE_2 _secretion. This consideration is very restrictive, since it is known that many processes are regulated by the balance between PGE_2 _and PGD_2_, which is also produced downstream of COX-2. Moreover, PGE_2 _secretion does not strictly reflect PGE_2 _production since it excludes PGE_2 _intracellular accumulation and/or degradation. Interestingly, two groups published their results in APC*^Min/+ ^*mice demonstrating on the one hand that the genetic deletion of mPGES-1, the terminal enzyme responsible for PGE_2 _synthesis, increased intestinal tumorigenesis [9], while on the other hand PGE_2 _treatment induced a raise in intestinal adenoma growth [4]. This apparent discrepancy suggests that PGE_2 _effects in intestinal tumorigenesis might not be restricted to those observed with extracellular provision.

Besides, we have observed in the glioblastoma [10] that the overexpression of mPGES-1 was correlated to a longer survival of patients. We have shown in glioblastoma that intracellular PGE_2 _induced a direct activation of the pro-apoptotic protein Bax, thereby inducing glioblastoma cells apoptosis [10], whereas extracellular PGE_2 _did not. The role played by PGE_2 _in cancer thus appears highly complex, whether in the whole tissue or even in isolated cancer cells. To gain understanding in the signalling of PGE_2 _in colon cancer cells, we focussed our work on the effect of intracellular PGE_2 _on the Bax-dependent apoptotic pathway.

## Methods

### Materials

Cell culture material was obtained from Gibco (Invitrogen, Cergy Pontoise, France). Unless mentioned, chemical products and reagents were obtained from Sigma (France).

Antibodies were purchased from indicated companies: COX-2 (Cayman, #160107), mPGES-1 (Cayman, #160140), actin (Chemicon, #MAB1501R).

15-PGDH inhibitor (CAY10397) was purchased from Cayman (#70130) (Interchim, France).

^3^H-PGE2 (0.1 μCi/μl) was purchased from Amersham Biosciences.

Immunoblots were quantified using the ImageJ software (NIH, USA).

Every experiment was repeated at least 3 independent times unless otherwise stated.

Statistical analyses were performed using the GraphPad software (San Diego, CA 92130 USA) (Student unpaired t-test, *: p < 0.05, **: p < 0.01).

### Patients

Patient materials as well as records (diagnosis, age, sex, date of death) were used with confidentiality according to French laws and recommendations of the French National Committee of Ethic. Tumor samples were collected from adult patients after surgical resection at the Department of anatomo-pathology of the Hospital of Nantes over the years 2002-2003. The clinical information of the patients is summarized in additional file 1 table SI, and additional file 2 table SII. Control tissue was obtained from normal colon tissue found at the periphery of the resected tumor.

### RT-PCR

Cells were washed twice in PBS, then total RNA was isolated using the RNAwiz (Ambion, Austin, TX, USA) according to the manufacturer's instructions with DNAse I treatment. After RNA quantification using the Nano Drop (Nano Drop ND-1000, Thermo Fisher Scientific, Waltham, MA, USA), the quality of the RNA was determined in an Agilent 2100 Bioanalyzer (Agilent, Palto Alto, CA, USA) using the Labchip RNA 6000 kit. A minimum RNA Integrity Number (RIN) value of 8 was required [11]. Total RNA (1 μg) was reverse transcribed in a final volume of 20 μl using the Superscript II kit (Invitrogen, France). Subsequently the cDNA was diluted to a final concentration of 20 ng/μl, for use in Q-PCR.

The PCR reaction contained 40 ng cDNA in a reaction volume of 25 μl, 1× Brilliant II SYBR Green Q-PCR master mix, 200 nM reverse and forward primers and 30 nM Sybr Green. Thermo-cycling conditions were 95°C for 10 min followed by 40 cycles at 95°C for 1 min, 60°C for 45 s and 72°C for 30 s. Gene expression values were normalized to housekeeping gene (GAPDH) and relative expression values were calculated based on the comparative ΔΔCT-method with adherent cells used as a reference for each cell type[12].

GAPDH: sense primer: 5'-GAAGGTGAAGGTCGGAGTC-3'

antisense primer: 5'-GAAGATGGTGATGGGATTTC-3'

COX-2: sense primer: 5'-CAGCCATACAGCAAATCC-3'

antisense primer: 5'-ATCCTGTCCGGGTACAAT-3'

mPGES-1: sense primer: 5'-AGGAAGACCAGGAAGTGC-3'

antisense primer: 5'-ACGACATGGAGACCATCTAC-3'

MRP4: sense primer: 5'-AAGTGAACAACCTCCAGTTCCA-3'

antisense primer: 5'-CCGGAGCTTTCAGAATTGAC-3'

15-PGDH: sense primer: 5'-AAGCAAAATGGAGGTGAAGGC-3'

antisense primer: 5'-TGGCATTCAGTCTCACACCAC-3'

### Cell culture and transfection

HCT-116 and HCT-116^Bax-/- ^cells (described in [13]) were grown in McCOY's 5A medium containing 10% fetal calf serum, 2 mM L-glutamine, 100 U/ml penicillin and 100 μg/ml streptomycin. SW1116 cells were grown in RPMI medium containing 10% fetal calf serum, 2 mM L-glutamine, 100 U/ml penicillin and 100 μg/ml streptomycin. The cells were transfected by a plasmid encoding for the sequence of mPGES-1 cDNA subcloned into pDEST12.2 vector (Invitrogen), or by the mock plasmid containing no coding sequence [10]. Plasmid DNA (5 μg) was introduced into 10^6 ^cells by electroporation (GenePulser, Bio-Rad) using 200 V/cm and 250 μF. Transfected cells were selected and further cultured in a medium containing 1 mg/ml G418. The mock-transfected cells were used as a control for the mPGES-1 transfected cells in the expression and viability experiments.

### Microinjection experiments

Microinjection was performed as described by Cartron *et al. *[14]. PGE_2 _was co-injected with a dextran coupled to a fluorochrome (Oregon Green, Molecular Probes). The instantaneous intracellular concentration of compounds achieved by the microinjection is about one tenth of the initial concentration in the injected solution. The percentage of fluorescent cells exhibiting morphological apoptotic features was evaluated every hour following PGE_2 _microinjection using an inverted fluorescent microscope (DMIRE2, Leica France).

### ^3^H-PGE_2 _internalisation assay.

HCT-116^Bax-/- ^cells were seeded in a 96-well culture plate the day before experiment. 5 μl ^3^H-PGE_2 _was added to the cells. Inhibitors of 15-PGDH (CAY10397, 15 μM) and MRP4 (ketoprofen, 1 μM [15]) were added in every other well. After the indicated incubation time (0 min, 30 min and 1 h), cells were rinsed and harvested. The amount of radiolabeled PGE_2 _present in the cells was quantified by beta-emission measurement (LS 6500 liquid scintillation counter, Beckman Coulter).

### Caspase activation assay

Total cell lysates were carried out with RIPA buffer (PBS, 1% NP-40, 0.5% Na-deoxycholate, 0.1% SDS, proteases inhibitor cocktail (Roche, Meylan, France)) and protein concentrations were measured by the Bradford technique. DEVDase activity was measured using the fluorometric CaspACE Assay System (Promega) and normalized to the sample protein concentration as described previously [10].

### Western blot

Total cell lysates were obtained with RIPA buffer and separated by SDS-PAGE. Proteins were transferred onto PVDF membranes by semi-dry transfer. Membranes were successively probed with the indicated antibodies and revealed by ECL with peroxidase-coupled secondary antibodies.

### Viability assays

SW1116 cells were plated the day before treatment. PGE_2 _(10 μM) was added to the culture medium. After 10 min, CAY10397 (15 μM) and ketoprofen (1 μM) were added, and cells were treated for 30 h. Cell death was then assessed by trypan blue staining.

## Results

### Heterogeneity of COX-2 and mPGES-1 expression in human colon cancer

**Figure 1:**
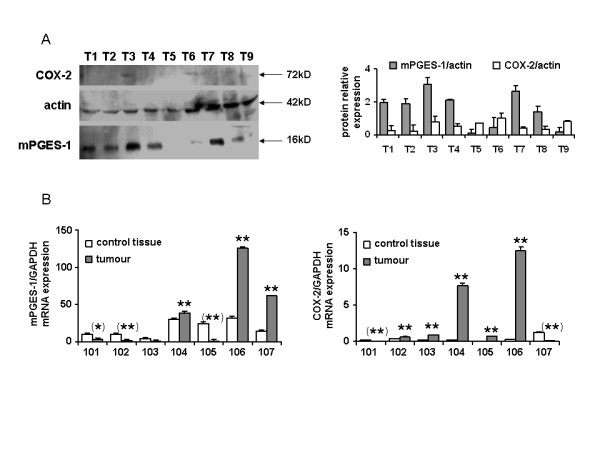
**Expression of mPGES-1 and COX-2 in human colon cancer tissues**. A: mPGES-1 and COX-2 expression were detected by western blot in 9 human colon cancer protein extracts. Band intensity was quantified and normalized to the corresponding intensity of actin in the sample. B: The expression of mPGES-1 and COX-2 transcripts was measured by RT-PCR in cDNA samples from 7 patients including paired normal and tumour colon tissues and normalized to GAPDH transcript. The significance of the difference between tumour and control tissue is indicated in brackets when the transcript is down-regulated in tumour.

The role played by PGE_2 _in cell survival/proliferation or in cell death is still highly debated. Given the observation we made in glioblastoma that mPGES-1 overexpression was correlated to an increased survival of patients, we studied the expression of COX-2 and mPGES-1 in nine human colon cancer samples (Figure 1A). mPGES-1 expression was very inconstant in the tumours, some expressing very high levels of the protein whereas mPGES-1 was hardly detectable in others. COX-2 expression was weakly detected in all the samples. We then measured mPGES-1 and COX-2 transcripts in seven additional human colon cancer samples and in the non cancerous corresponding tissue by qPCR (Figure 1B). Similarly, mPGES-1 expression appeared very inconstant whereas COX-2 was regularly overexpressed in cancer tissue compared to control (p < 0.003 in 5 out of 7 patients), even if the mRNA relative expression level was low (see mPGES-1, left graph, for comparison). Of note, in two out of three tumours overexpressing mPGES-1, COX-2 was largely overexpressed. In our hands, it thus seems that mPGES-1 was not always up-regulated in colon cancers.

### Effect of mPGES-1 overexpression in human colon cancer cell lines

**Figure 2:**
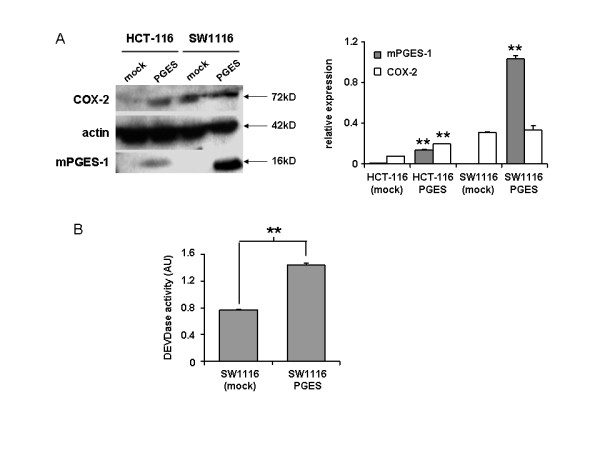
**Expression of mPGES-1 in colon cancer cell lines**. A: mPGES-1 and COX-2 expression were detected by western blot in the colon cancer lines HCT-116 and SW1116, either mock-transfected or overexpressing mPGES-1 (HCT-116/PGES and SW1116/PGES respectively). The bands intensity was quantified and normalized to the corresponding intensity of actin in the sample (graph). B: mock-transfected SW1116 cells and SW1116 cells overexpressing mPGES-1 were submitted to UV-B irradiation and DEVDase activity was measured in the cell lysate after 30 h as described earlier [10].

We have previously shown in glioblastoma primary cultures that mPGES-1 exogenous overexpression sensitized the cells to apoptosis [10]. We thus considered if this observation could be reproduced in human colon cancer cells *in vitro*, when isolated from the stroma. We first analysed the expression of mPGES-1 and COX-2 in two model human colon cancer cell lines (HCT-116 and SW1116). As shown in Figure 2A, mPGES-1 expression was hardly detectable in both cell lines (Figure 2A right, lanes 1 and 3). In contrast, COX-2 was abundantly expressed in SW1116 cells, but not in HCT-116 cells. HCT-116 cells have often been presented as COX-2 deficient cells, whereas they rather seem to constitutively express COX-2, unlike non-cancer cells, even at lower level than other cell lines. We induced mPGES-1 overexpression in both cell lines by plasmid transfection. Of note, two rounds of transfection were necessary to induce a steady expression of mPGES-1 in HCT-116 cells. As we had observed earlier in glioblastoma cells, the modulation of mPGES-1 expression had an effect on the expression of COX-2. Surprisingly, COX-2 expression was increased in HCT-116 cells overexpressing mPGES-1 whereas it was not significantly modified in SW1116 transfected cells (Figure 2A left, lanes 2 and 4). We verified that the transfection of mPGES-1 in these two cell lines and in two additional colon cell lines (HCT-8 and HT29) was accompanied by an increase in intracellular PGE_2 _concentration (additional file 3 figure S1A). Since the expression of COX-2 was not modified by the transfection in SW1116 cells, we used these cells for the rest of the experiment. SW1116 cells were subjected to UV-B irradiation to induce apoptosis (25 J/cm^2^, 10 min). The specific caspase-3 activity was measured in the cell lysate after 30 hours. As shown in Figure 2B, mPGES-1 overexpression led to a higher caspase-3 activity, attesting to a higher sensitivity of the cells to apoptosis induction. Of note, we measured a constitutive caspase-3 activity in all the four mPGES-1-transfected cells, even if this activity was very low and was not associated to a significant cell death (additional file 3 figure S1B).

### Effect of PGE_2 _microinjection in colon cancer cell

**Figure 3:**
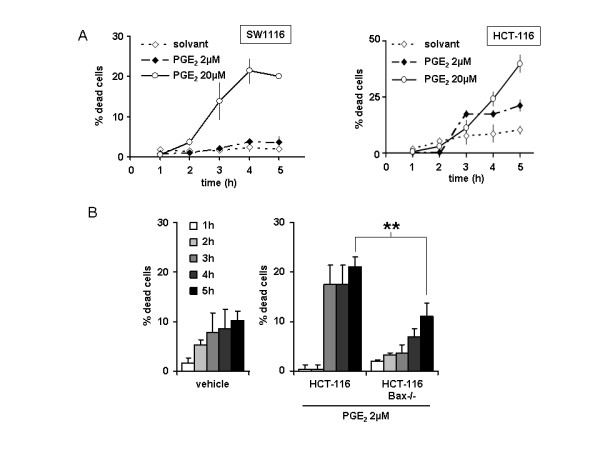
**Effect of PGE_2 _cytoplasmic microinjection**. A: SW1116 and HCT-116 cells were microinjected with PGE_2 _as described in the material and methods section. The graph represents the amount of dead cells among the microinjected population over the time. B: PGE_2 _microinjection was performed in HCT-116 and HCT-116^Bax-/- ^cells as in A.

The conflicting observations that extracellular PGE_2 _promotes colon cancer cells survival and proliferation [3,4,8] while mPGES-1 overexpression increases apoptosis in colon cancer cells (Figure 2B andadditional file 3 figure S1B) prompted us to study the effect of PGE_2 _microinjection directly into the cytoplasm of colon cancer cells. The treatment of the four colon cancer cell lines with PGE_2 _extracellular concentrations ranging from 0.1 μM to 100 μM induced no more than 10% cell death compared to control cells. Yet HCT-116 cells exhibited the highest variation coefficient in cell death following to PGE_2 _extracellular treatment (additional file 4 figure S2). As shown in Figure 3A, PGE_2 _microinjection induces a significant cell death within 5 hours in both SW1116 and HCT-116 cell lines, whereas SW1116 cells are only sensitive to the highest concentration injected (left). Of note, the microinjection of a 20 μM PGE_2 _solution results in a transient intracellular increase of about 2 μM, which is consistent with the intracellular levels we have previously measured during apoptosis induction (unpublished results). Consistent with the direct effect of PGE_2 _on Bax activation we have previously demonstrated, we observed that the cell death induced by PGE_2 _in colon cancer cells was dependent on Bax expression. Indeed, Bax-deficient HCT-116^Bax-/- ^cells appeared resistant to PGE_2 _microinjection (Figure 3B).

### Effect of PGE_2 _intracellular accumulation induced by pharmacological agents.

**Figure 4:**
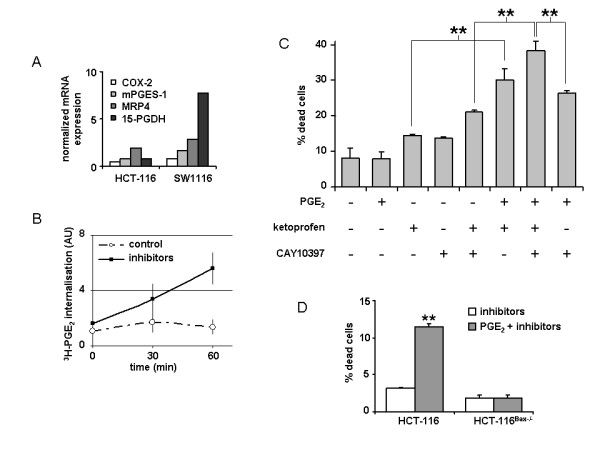
**Effect of constraint PGE_2 _internalisation on cell viability**. A: the normalized expression of COX-2, mPGES-1, MRP4 and 15-PGDH transcripts were measured in HCT-116 and SW1116 cells. The graph shown is representative of 2 independent experiments. B: the internalisation of ^3^H-PGE_2 _added in the medium in HCT-116^Bax-/- ^cells is shown. C: SW1116 cells were treated with the indicated combination of extracellular PGE_2 _and inhibitors and cell death was assessed by Trypan blue exclusion. D: HCT-116 and HCT-116^Bax-/- ^cells were treated by 10 μM PGE_2 _with or without both inhibitors and cell death was assessed by Trypan blue exclusion.

Since PGE_2 _microinjection was able to induce colon cancer cell death, whereas extracellular PGE_2 _could not, we tried to force PGE_2 _cytoplasmic accumulation by pharmacological means. PGE_2 _intracellular concentration is the result of several regulated processes, among which are its synthesis, its enzymatic degradation through the activity of 15-PGDH (15-prostaglandin E_2 _dehydrogenase), and its membrane transport, mainly through the export protein MRP4. We thus analysed the level of the transcripts of the synthesis enzymes COX-2 and mPGES-1 and also those of 15-PGDH and MRP4 in both cell lines HCT-116 and SW1116 (Figure 4A). It was noticeable that SW1116 cells expressed a high level of 15-PGDH compared to HCT-116, possibly explaining the significant difference of sensitivity to PGE_2 _microinjection (Figure 3A). Ketoprofen was used, at a non-COX-2 inhibiting dose, to block the MRP4-mediated exportation of PGE_2 _[15], while CAY10397 was used to inhibit PGE_2 _enzymatic degradation by 15-PGDH. Treating the cells with both drugs caused a significant cytoplasmic accumulation of ^3^H-PGE_2_, as measured in the resistant HCT-116^Bax-/- ^cells (Figure 4B, black squares), whereas ^3^H-PGE_2 _remained extracellular in the absence of MRP4 inhibition (Figure 4B, open circles). Given this result, we treated SW1116 cells with extracellular PGE_2 _and with various combinations of the inhibitors described and measured cell death after 30 hours by Trypan blue exclusion. As shown in Figure 4C, the combination of both inhibitors induced a significant cell death even in the absence of exogenous PGE_2_. However, cell death increased from 21 to 38% when PGE_2 _was added. A significant, yet slightly lower cell death induction was observed when either CAY10397 or ketoprofen was omitted, indicating that both PGE_2 _enzymatic degradation and exportation participate in the survival of these. We verified in SW1116 cells that the treatment applied effectively induced an increase in PGE_2 _intracellular concentration and that caspase 3 was activated, testifying apoptotic cell death (additional file 5 figure S3A). Of note, the treatment described above also enabled PGE_2 _intracellular accumulation and cell death in the three additional colon cell lines used (additional file 5 figure S3B); besides, we confirmed the singularity of HCT-116 cells compared to the three other cell lines since PGE_2 _internalisation was measured even in the absence of the inhibitors, making their sensitivity to extracellular PGE_2 _clearer. We also verified, using the HCT-116 cell line and its Bax-deficient counterpart (HCT-116^Bax-/-^), that the cell death induced by the combination of extracellular PGE_2 _and the inhibitors described above was Bax-dependent (Figure 4D).

## Discussion

An extensive amount of data point out that COX-2 and its product PGE_2 _are actors of cancer promotion and progression. This point has been supported by the fact that the use of COX inhibitors reduces the incidence of several cancers, among which colorectal cancer [1]. Mechanistically, it has been established that PGE_2_, one of the products of COX-2 activity, could activate several pathways implicated in cancer, namely apoptosis evasion, cell proliferation, migration and angiogenesis. The majority of these effects are mediated through G-coupled EP receptors (EP1-4) [2]. Nevertheless, conflicting results tend to demonstrate that PGE_2 _is much more versatile than what was initially thought.

Besides, caution should be used when considering COX inhibitors as "anti-PGE_2_" compounds. The inhibition of COX-2, even with the use of selective COX-2 inhibitors, definitely has larger consequences than a decrease in PGE_2 _synthesis since several prostaglandins arise from COX-2 activity. For instance, Thoren and Jakobsson [16] demonstrated that COX-2 inhibitors had a various ability to inhibit mPGES-1 activity. As a consequence, COX-2 inhibitors not only modulate COX-2 activity but also the potential coupling of COX-2 and mPGES-1 activity; they consequently not only modulate PGH_2 _production but also the ratio between the PGH_2 _products, among which PGE_2 _and PGD_2_, which are known to exert opposite effects on Bax activation [17].

Given the multiplicity of the physiological functions of prostaglandins and the very subtle regulatory processes which can hardly be predicted in the whole, we need a deeper understanding of the pathways in which the products of COX are implicated in cell signalling, in the tissue and in the body, before safely using NSAIDs as anti-cancer therapeutic adjuvants. More disturbing is the observation that the anti-proliferative effects of COX-2 inhibitors on cancer cells have also been demonstrated in COX-2-deficient cells [18-21], suggesting that the role of COX-2 and its product PGE_2 _in cancer might have been overvalued based on the effects of pharmacological COX-2 inhibitors.

We show here that the overexpression of mPGES-1, the enzyme responsible for PGE_2 _synthesis downstream of COX-2, sensitizes isolated colon cancer cells to apoptosis in vitro. We also demonstrate that cell death can be induced in colon cancer cells by increasing the intracellular content in PGE_2_, either through direct microinjection or through the inhibition of PGE_2 _intracellular exportation and degradation, provided the cells express the protein Bax. Indeed, taking into account the data of Reid and colleagues [15], which showed that several NSAIDs exert an inhibitory activity on MRP4 at concentrations inferior to those used for COX inhibition, we showed that ketoprofen could induce a PGE_2_-dependent cell death, even if MRP4 inhibition might inhibit the efflux of other compounds from the cells; this could partly explain the disastrous cardiac side effects observed during long-term NSAID treatments. Similarly, we did not explore the consequence of 15-PGDH inhibition on the concentration of other prostaglandins, but we showed that the cell death was considerably increased by the adding of PGE_2 _in the cell culture, demonstrating that PGE_2 _was at least partly responsible for the apoptosis induced. These results are consistent with what we have previously described in glioblastoma [10]. To our comprehension, our results also bring a possible explanation to some of the conflicting results observed between extracellular PGE_2 _treatments and modulations of PGE_2 _production (see [4] and [9] for example). With the care to be as representative as possible for colon cancers, our in vitro work was realised with four colon cancer cell lines, two of which presented LOH (SW1116 and HT29) whereas the other two were classified MSI-positive (HCT-116 and HCT-8)[22,23].

What could be the rationale of these ambiguous properties exhibited by PGE_2_? An attractive concept was recently described by Li and colleagues [24]. They report that executive caspases, key players of apoptotic cell death, are necessary for wound healing. The activation of these caspases in injured cells is responsible for PGE_2 _synthesis and exportation. When released at the wounded site, PGE_2 _stimulates stem cells proliferation and tissue regeneration. PGE_2 _might thus be regarded as a danger signal emerging from dying cells. Our understanding of the mechanism is that newly produced, intracellular PGE_2 _is able to sensitize the cells to death through the activation of the apoptotic protein Bax. In the cells where the death signals overwhelm the resistance capacities, caspases are activated and apoptosis occurs. Meanwhile, PGE_2 _production and release in the environment is increased; PGE_2 _thus exerts its antiapoptotic, proliferative and migratory role on the neighbouring cells through the EP receptors pathway. In the context of a tumour, the surviving cells become more resistant to a subsequent insult. PGE_2 _activity in tumour cells would thus follow a two-step mechanism: first, intracellular PGE_2 _participates in apoptotic cell death; second, secreted PGE_2 _has an autocrine or paracrine protective and stimulating activity, respectively on the producing cell if the integration of death signals is compatible with survival, or on the neighbouring cells if cell death is induced, with an amplification loop in PGE_2 _production through executive caspases activity. The important consequence of this mechanism is that PGE_2 _exportation from cancer cells is the most detrimental determinant in the role played by PGE_2 _in tumour progression. An alternative to COX-2 inhibitors as adjuvant anti-cancer therapies might thus be the use of drugs inhibiting PGE_2 _efflux from the cancer cells. The potential of MRP4 inhibitors in enhancing classical therapies would thereby be worth questioning.

## Conclusions

Our present work demonstrates that intracellular PGE_2 _can exert a pro-apoptotic, Bax-dependent apoptosis in colon cancer cell lines in vitro. We thereby bring an additional level of complexity in the highly complex role played by PGE_2 _in colon cancer progression. We also suggest that MRP4 inhibition might be a valuable adjuvant strategy to colon cancers treatments.

## Abbreviations

NSAIDs: non steroidal anti-inflammatory drugs; PGE_2_: prostaglandin E_2_; PGD_2_: prostaglandin D2; COX-2: cyclooxygenase 2; mPGES-1: microsomal prostaglandin E_2 _synthase-1; 15-PGDH: 15-hydroxyprostaglandin dehydrogenase.

## Competing interests

The authors declare that they have no competing interests.

## Authors' contributions

LL participated to the design of the study, to the collection and analysis of PGE_2 _internalisation data and drafted the manuscript. FP collected and analysed qPCR data. CB participated to the collection and analysis of data. JM and FMV participated to the design and coordination of the study and helped to draft the manuscript. CO helped to the design of the study, collected and analysed data and helped to draft the manuscript. All authors read and approved the final manuscript.

## Pre-publication history

The pre-publication history for this paper can be accessed here:

http://www.biomedcentral.com/1471-2407/11/153/prepub

## Supplementary Material

Additional file 1 **table SI**. clinical information of the first set of patients. Click here for file

Additional file 2 **table SII**. clinical information of the second set of patients. Click here for file

Additional file 3 **figure S1**. PGE_2 _intracellular measurement and DEVDase activity in 4 mPGES-1 transfected colon cell lines. Click here for file

Additional file 4 **figure S2**. effect of extracellular PGE_2 _on the 4 colon cancer cell lines viability. Click here for file

Additional file 5 **figure S3**. extracellular PGE_2 _internalisation in the 4 colon cancer cell lines. Click here for file
