# Supplementary figures and images for: Increase in intracellular PGE2 induces apoptosis in Bax-expressing colon cancer cell
Source: BMC Cancer. 2011 Apr 27;11:153. doi: 10.1186/1471-2407-11-153 (PMC3097003; doi:10.1186/1471-2407-11-153)

**Table S1:** clinical information of the first set of patients.


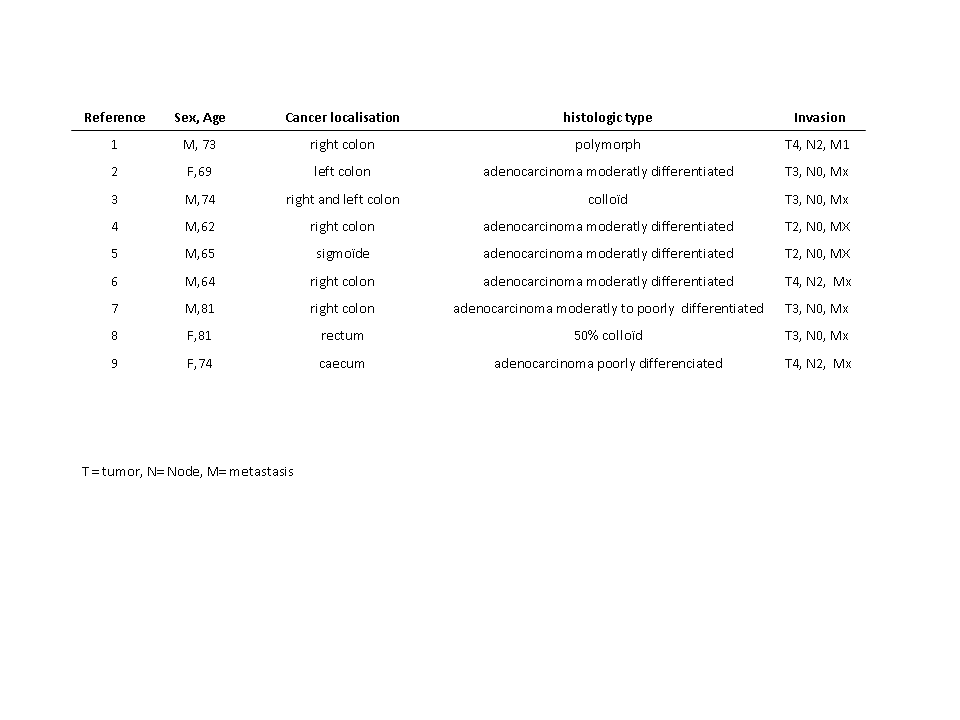

Supplement: Additional file 1 — table SI. clinical information of the first set of patients. [file 1471-2407-11-153-S1.DOC]

**Table S2:** clinical information of the second set of patients.


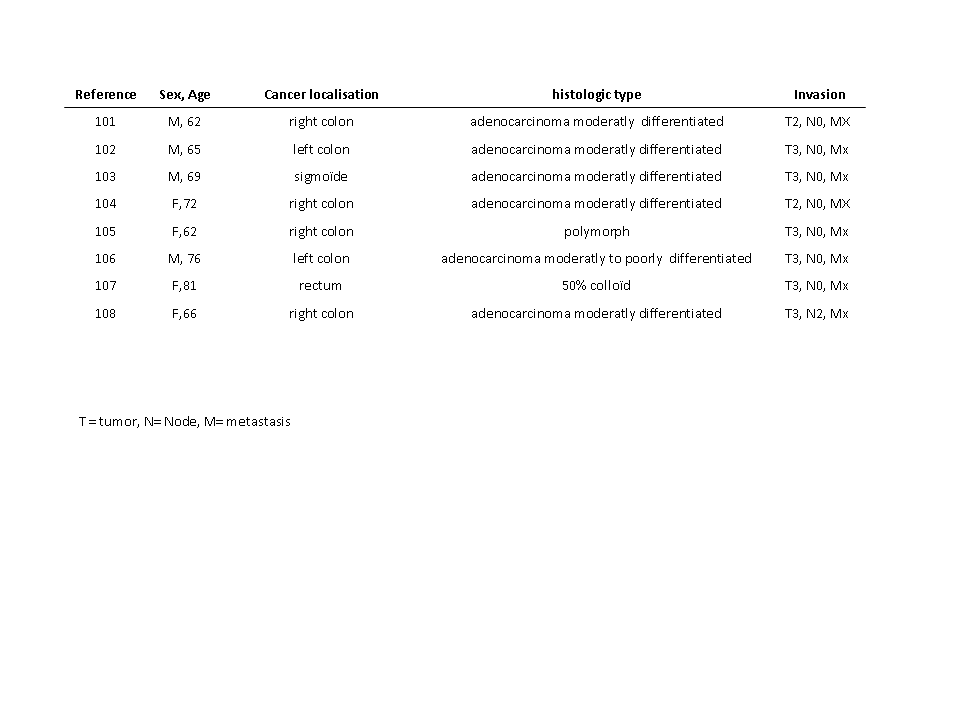

Supplement: Additional file 2 — table SII. clinical information of the second set of patients. [file 1471-2407-11-153-S2.DOC]
