# Supplementary material for: Increase in intracellular PGE2 induces apoptosis in Bax-expressing colon cancer cell
Source: BMC Cancer. 2011 Apr 27;11:153. doi: 10.1186/1471-2407-11-153 (PMC3097003; doi:10.1186/1471-2407-11-153)

**Figure S1**: A) mPGES-1 overexpression was induced by transfection in the four colon cancer cell lines indicated. Intracellular PGE_2_ was measured using Amersham PGE_2_ Biotrak EIA system (#RPN222) according to the manufacturer. Protein concentration was measured in the cell lysate and PGE_2_ measurements were normalized to the protein concentration. The graphs represent the mean from four experiments (*:p<0.05, **:p<0.01). B) DEVDase activity was measured in cell lysates obtained from the transfected cells indicated. The activity was normalized to the protein concentration. The graphs represent the mean from three experiments (**:p<0.01).


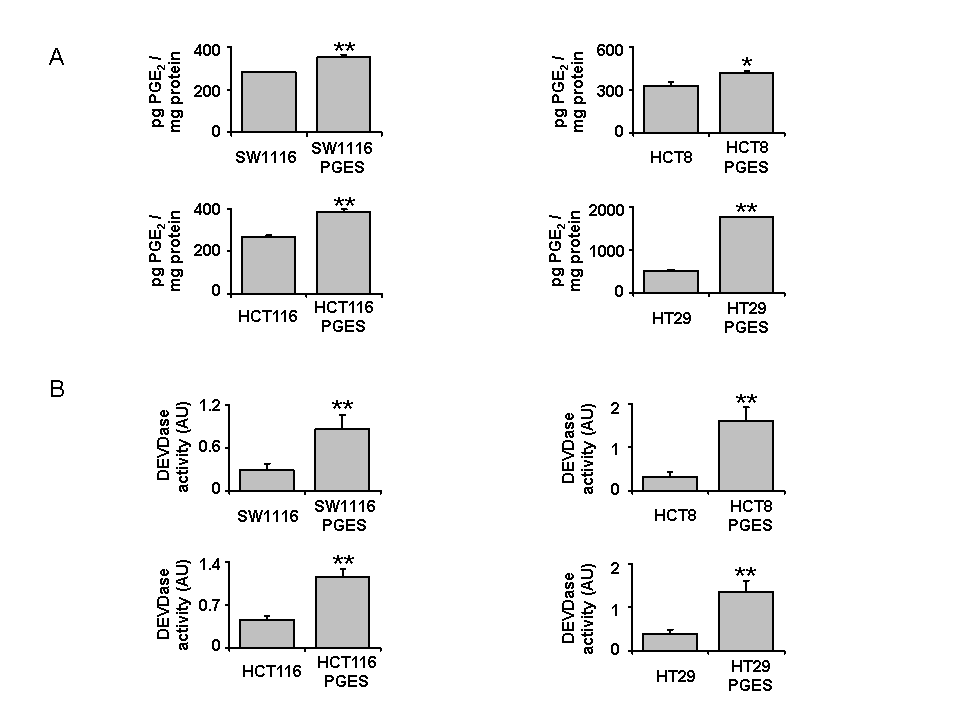

Supplement: Additional file 3 — figure S1. PGE2 intracellular measurement and DEVDase activity in 4 mPGES-1 transfected colon cell lines. [file 1471-2407-11-153-S3.DOC]
