# Supplementary material for: Increase in intracellular PGE2 induces apoptosis in Bax-expressing colon cancer cell
Source: BMC Cancer. 2011 Apr 27;11:153. doi: 10.1186/1471-2407-11-153 (PMC3097003; doi:10.1186/1471-2407-11-153)

**Figure S2**: PGE_2_ was added to the cell culture at the indicated concentration to the four cell lines for 24h. Cell death was assessed and the coefficient of variation (%cv) versus control cells was determined.


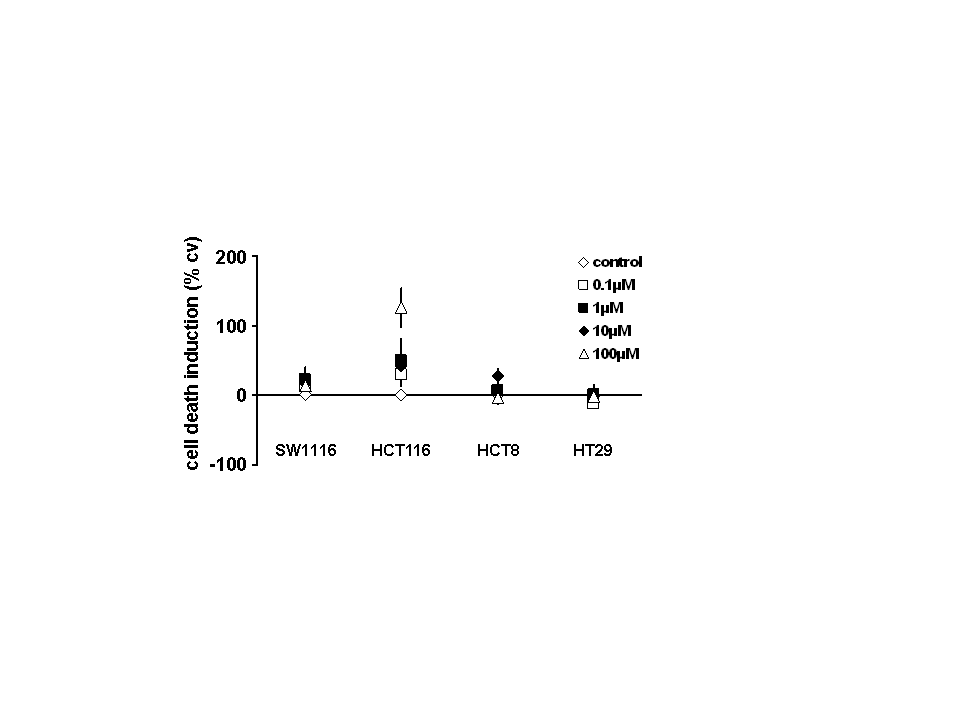

Supplement: Additional file 4 — figure S2. effect of extracellular PGE2 on the 4 colon cancer cell lines viability. [file 1471-2407-11-153-S4.DOC]
