# Supplementary material for: Increase in intracellular PGE2 induces apoptosis in Bax-expressing colon cancer cell
Source: BMC Cancer. 2011 Apr 27;11:153. doi: 10.1186/1471-2407-11-153 (PMC3097003; doi:10.1186/1471-2407-11-153)

**Figure S3**: A) SW1116 cells were treated by 10µM PGE_2_ with or without MRP4 and 15-PGDH inhibitors as described in material and methods. The amount of intracellular PGE_2_ was measured as in supplementary figure 1 after 24 hours (left). The DEVDase activity was measured as described above in the cells treated by the combination of PGE_2_ and inhibitors (right). B) The three additional cell lines were treated as described in A). Intracellular PGE_2_ was measured as described above (left). Cell death was assessed after a 30 hours treatment (right).


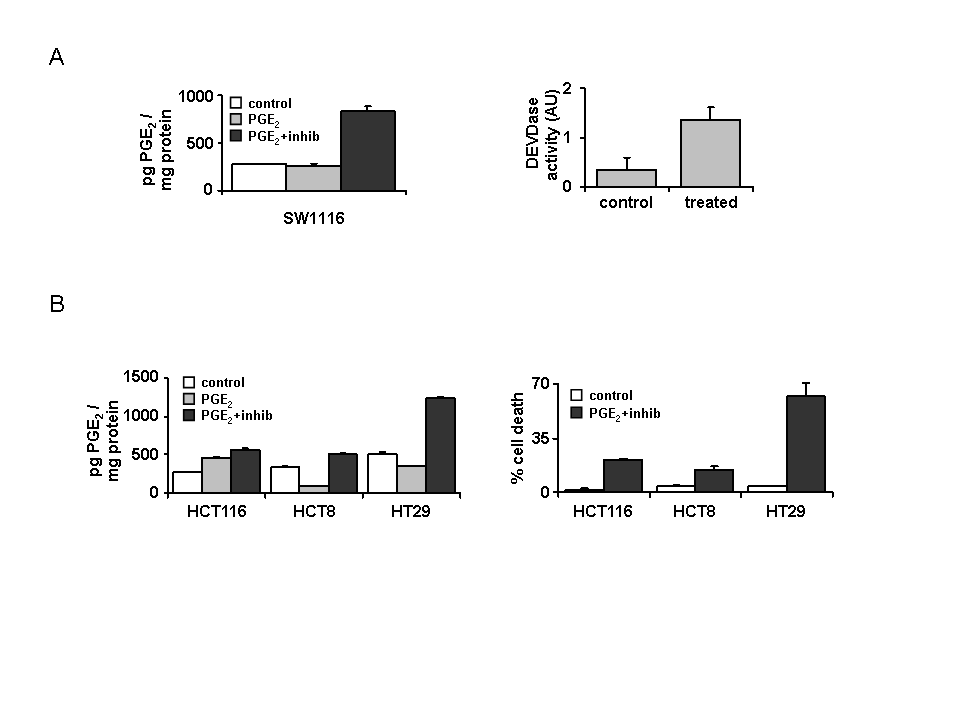

Supplement: Additional file 5 — figure S3. extracellular PGE2 internalisation in the 4 colon cancer cell lines. [file 1471-2407-11-153-S5.DOC]
